# Feasibility and Preliminary Effectiveness of a Tele-Prehabilitation Program in Esophagogastric Cancer Patients

**DOI:** 10.3390/jcm9072176

**Published:** 2020-07-09

**Authors:** Elise Piraux, Gilles Caty, Gregory Reychler, Patrice Forget, Yannick Deswysen

**Affiliations:** 1 Pôle de Neuro Musculo Skeletal Lab, Pôle de Pneumologie, ORL & Dermatologie, Institut de Recherche Expérimentale et Clinique, Clinical Neuroscience, Institute of Neurosciences, Université Catholique de Louvain, 1200 Brussels, Belgium; 2 Pôle de Neuro Musculo Skeletal Lab, Institut de Recherche Expérimentale et Clinique, Clinical Neuroscience, Institute of Neurosciences, Université Catholique de Louvain, Service de Médecine Physique et Réadaptation, Cliniques Universitaires Saint-Luc, 1200 Brussels, Belgium; gilles.caty@uclouvain.be; 3 Pôle de Pneumologie, ORL & Dermatologie, Institut de Recherche Expérimentale et Clinique, Université catholique de Louvain, Haute Ecole Léonard de Vinci, PARNASSE-ISEI, Secteur de kinésithérapie, Service de Pneumologie, Cliniques Universitaires Saint-Luc, 1200 Brussels, Belgium; gregory.reychler@uclouvain.be; 4 Institute of Applied Health Sciences, Epidemiology Group, University of Aberdeen, NHS Grampian, Department of Anaesthetics, Aberdeen AB25 2ZD, UK; forgetpatrice@yahoo.fr; 5 Upper Gastrointestinal Surgery Unit, Cliniques Universitaires Saint-Luc, 1200 Brussels, Belgium; yannick.deswysen@uclouvain.be

**Keywords:** exercise, prehabilitation, preoperative care, tele-rehabilitation, oesophagogastric cancer

## Abstract

Tele-rehabilitation provides better access to healthcare services and optimizes exercise adherence. However, its feasibility and effectiveness are unknown in the preoperative period in esophagogastric cancer patients. We aimed to assess the feasibility and the preliminary effects of a “tele-prehabilitation” program in esophagogastric cancer patients requiring surgery. Enrolled participants performed an internet-based tele-prehabilitation including aerobic, resistance and inspiratory muscle training over 2–4 weeks. The primary outcome was feasibility, measured in terms of recruitment, retention and attendance rates, adverse events and patient satisfaction. Secondary outcomes (functional exercise capacity, fatigue, quality of life, anxiety and depression) were assessed at baseline, presurgery, and 4 and 12 weeks postsurgery. Among the 24 eligible subjects, 23 were enrolled, 22 performed the intervention and 15 completed the study. Recruitment and retention rates were both 96%. Attendances to aerobic and resistance sessions and inspiratory muscle training were 77% and 68%, respectively. No adverse events occurred, and the satisfaction was excellent. After prehabilitation, participants significantly improved fatigue (*p* = 0.039), quality of life (*p* = 0.009), physical well-being (*p* = 0.034), emotional well-being (*p* = 0.005) and anxiety (*p* = 0.044). This study demonstrated the feasibility of a tele-prehabilitation in esophagogastric cancer patients undergoing surgery, with a high recruitment rate, retention rate and satisfaction, a good attendance to exercise sessions and no exercise-related adverse events.

## 1. Introduction

Worldwide, esophageal and gastric cancers are the seventh and fifth most common cancers and the sixth and third leading causes of cancer death, respectively [1]. Surgical resection is currently the main curative treatment for localized or locally advanced esophagogastric cancers with or without a neoadjuvant treatment [2,3].

Despite progress in surgical techniques, these surgical resections remain associated with a high incidence of perioperative morbidity and mortality [4]. Esophagogastric resections also have short- and long-terms side effects including impaired functional status, fatigue, quality of life (QoL) and psychological distress [5,6,7,8]. Many publications have described serious concerns regarding patients’ preoperative physical status and its association with postoperative complications and mortality [9,10,11]. Additionally, this status can be affected by advanced age and neoadjuvant treatment [12,13].

In recent years, there has been a growing interest in optimizing the preoperative period to increase a patient’s physical capacity, with the intention to improve postoperative outcomes and accelerate recovery [14]. Although this preoperative intervention—“prehabilitation”—has been shown to be effective in improving preoperative physical fitness and postoperative outcomes in patients with various types of cancer [15,16], there are only limited, albeit encouraging, results on the feasibility and effectiveness of prehabilitation in esophagogastric cancer patients [17,18,19]. Minnella et al. showed that a multimodal prehabilitation intervention improved functional capacity before and after surgery compared to the control group, but no difference was found concerning postoperative complications, length of hospital stay, emergency department visits or readmission rates [17]. Other studies reported a reduction in postoperative pulmonary complications after physical prehabilitation in cancer patients undergoing esophagectomy, while an increase in muscle strength was observed in elderly sarcopenic patients with gastric cancer [18,19,20].

Although a preoperative physical program seems to provide benefits, engaging these patients in a prehabilitation program can be a challenge, especially for the most complex ones. Indeed, cancer diagnosis implies already many consultations or examinations at the hospital. Adding visits for exercise sessions can become a burden for the patients who are often elderly and in poor physical condition. The main barriers reported by cancer patients to participate in a prehabilitation program are transport-related problems [21], suggesting that a home-based exercise program would be more appropriate to increase accessibility and avoid additional transportation for patients.

Tele-rehabilitation, a treatment delivered via broad-reach modalities through different technological means [22], could be an effective way to overcome these barriers to exercise. It provides easier access to health care services and could, therefore, maximize exercise adherence. The feasibility and effectiveness of tele-rehabilitation have been shown in cancer patients [23] and surgical patients [24]. However, the feasibility of a preoperative “tele-prehabilitation” has not yet been investigated in esophagogastric cancer patients requiring surgery.

Accordingly, the primary objective of this study was to determine the feasibility of a tele-prehabilitation program for esophagogastric cancer patients requiring surgery. Secondary objectives were to investigate the evolution of the functional exercise capacity, cancer-related fatigue (CRF), QoL, anxiety and depression before and after the tele-prehabilitation program, with a postoperative follow-up.

## 2. Materials and Methods

### 2.1. Ethics

Written informed consent was obtained from all included participants. The protocol of this study was approved by the regional Ethics Committee of the Cliniques universitaires Saint-Luc and Université catholique de Louvain (decision no. 2017/05OCT/469 on 8 January 2018) and was conducted in accordance with the Declaration of Helsinki. This study was previously registered at clinicaltrials.gov (NCT03418298).

### 2.2. Design and Study Sample

Participants were prospectively recruited from February 2018 to March 2019 in the upper gastrointestinal surgery unit in Cliniques universitaires Saint-Luc (Brussels, Belgium). Participation in the study was proposed to all potentially eligible patients. Eligible criteria included subjects with a diagnosis of esophageal or gastric cancer planned for surgery with or without neoadjuvant treatment and aged over 18 years. Subjects were not eligible if they were inoperable or operated on emergency. They were excluded if they had any conditions that contraindicate or limit exercise, no internet access or inability to use internet. These reasons for exclusion were prospectively documented. Included patients with the presence of signs or symptoms and/or known cardiovascular, metabolic, or renal disease had to have the approval of a health care professional to engage in the exercise program [25].

### 2.3. Intervention

Internet-based program: An online tele-prehabilitation platform was set up using the Virtuagym© fitness application (Virtuagym, Amsterdam, Netherlands). After baseline assessment, patients received information on how to use the platform by a physiotherapist as well as an explanation form including how to navigate the website. Each patient had his/her own user account on which the physiotherapist added the program to the patient’s schedule. The program was personalized based on the patient’s initial functional capacity. During this individual session, the physiotherapist showed the patients the complete exercise program and provided advice on how to carry out the exercises safely. On the account, the patient found an exercise schedule, a description of each exercise session and a tab to send an e-mail to the physiotherapist. The website was composed of a set of exercise videos and was available on computer, tablet or mobile phone. Patients were asked to follow the instructions and perform the exercises presented on the screen. After each exercise session, patients were asked to validate the exercises performed and to report adverse events.

Prehabilitation program: The tele-prehabilitation program started 2 or 4 weeks before surgery, depending on the treatment, and ended the day before surgery. The 2 week prehabilitation was provided to patients whose cancer was treated through primary surgery, while the 4 week prehabilitation was given to patients undergoing a neoadjuvant treatment before surgery. It included aerobic and resistance training 3 times a week and inspiratory muscle strengthening 5 times a week. Exercise sessions were unsupervised, but a physiotherapist made weekly phone calls to answer questions from the patients, motivate them to complete the exercises and adapt the program if necessary. Aerobic training was performed for 30 min at an intensity of 65–74% of maximum heart rate (HRmax = 220−age) controlled with a heart rate monitor (Polar, FT7, Electro Oy, Kempele, Finland). Patients chose an activity such as walking, cycling or rowing according to their preferences and capabilities. The progressive resistance training consisted of 8–10 weight-bearing exercises targeting major muscle groups of the trunk, upper limbs and lower limbs for an average of 30 min. Subjects performed 1 to 4 sets of 8–12 repetitions at a moderate intensity, rated as 4–6 on rated perceived exertion (range 0–10 on the modified Borg scale). The volume of resistance exercises varied over the course of the sessions. Inspiratory muscle training (IMT) was undertaken with an inspiratory threshold-loading device (Threshold IMT; Philips Respironics, Inc., Murrysville, PA, USA) for 15 min. Patients were asked to maintain a respiratory rate of 15 to 20 breaths per minute. The inspiratory resistance of the IMT started at 30% of the maximal inspiratory mouth pressure measured at baseline with a lung function test and was gradually increased by 5% if the rate of perceived exertion on the Borg scale remained under 5 [26,27].

### 2.4. Outcome Measures

The primary outcome was the program feasibility assessed by recruitment rate, retention rate, attendance to exercise sessions, exercise-related adverse events and patient satisfaction. Recruitment rate was defined as the ratio of the number of the recruited patients to the number of eligible patients. Retention rate was defined as the percentage of patients who complete the tele-prehabilitation program out of the number of enrolled patients. Attendance to exercise sessions was defined as the percentage of the sessions completed out of the actual number of sessions prescribed. The completion of the sessions, self-reported by the patient in the fitness application, was used to calculate the attendance. Safety was assessed by identifying exercise-related adverse events or symptoms. Patient satisfaction toward the prehabilitation was reported by a survey on satisfaction developed by the authors and completed at the end of prehabilitation. This survey included eight items about satisfaction of the tele-prehabilitation program with five levels of satisfaction ranging from “very satisfied” to “not at all satisfied”. In addition, patients were asked to rate their overall satisfaction with participation in the program on a 10-point scale.

Secondary outcomes included functional exercise capacity, CRF, QoL, anxiety and depression and were recorded at baseline (at the beginning of the prehabilitation program) (T0), presurgery (at the end of prehabilitation, i.e., the day before surgery) (T1), 4 weeks postsurgery (T2) and 12 weeks postsurgery (T3). Functional exercise capacity was evaluated by the 6 min walk test (6MWT). The 6 min walk distance (6MWD) covered in a 30 m hallway was recorded [28]. The Minimal Clinically Important Change (MCID) is a change of 20 m [29]. CRF was evaluated using the Functional Assessment of Chronic Illness Therapy-Fatigue Scale (FACIT-F) [30]. The FACIT-F comprises 13 items using a 5-point Likert scale ranging from 0 (not at all) to 4 (very much); the higher the score, the lower the fatigue. The MCID is 3 points [31]. Cancer-specific QoL was assessed with the Functional Assessment of Cancer Therapy-General (FACT-G) [32]. The FACT-G contains 27 items divided into four categories: Physical well-being (PWB), social/family well-being (SWB), emotional well-being (EWB), and functional well-being (FWB). Each item is rated from 0 (not at all) to 4 (very much) with a total score ranging from 0 to 102; the higher the score, the better the QoL. The MCID for FACT-G is equal to 4 points [31]. The Hospital Anxiety and Depression Scale (HADS) was used to assess anxiety and depression [33]. This is a self-administered scale of 14 items, divided into two subscales of seven items (Anxiety: HADS-A; Depression: HADS-D), each ranging from 0 to 3. Two sub-scores (HADS-A and HADS-D) are calculated and vary from 0 to 21, with higher scores indicating more anxious/depressive symptoms [33].

### 2.5. Statistical Analysis

Statistical analysis was performed with SigmaStat 13.0 (WPCubed GmbH, Munich, Germany) on an intention-to-treat basis [34]. A *p*-value of less than 0.05 was considered statistically significant. The normality of the data distribution was verified using the Shapiro–Wilk test. All the analyzed variables were normally distributed, and parametric tests were performed. Descriptive statistics were reported for participants and presented as means ± standard deviation (SD) for continuous variables and absolute frequencies (percentage) for categorical variables. Changes in test scores over the four time points were analyzed using the repeated-measures analysis of variance. Holm–Sidak post hoc corrections were used to perform adjustment for multiple groupwise comparisons. Data are presented as mean ± SD.

## 3. Results

### 3.1. Feasibility

**Figure 1:**
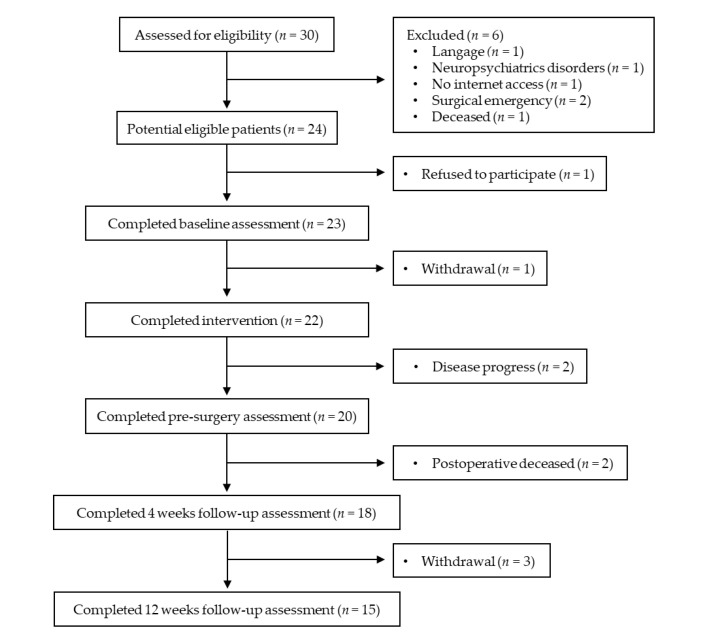
Participant flow chart.

The participant flow diagram is presented in Figure 1. Of the 30 patients assessed for eligibility, six were ineligible. Among the 24 eligible subjects, one refused to participate (recruitment rate of eligible patients = 96%). Of the 23 enrolled subjects, one withdrew during the exercise program (retention rate = 96%). Two subjects were excluded at the end of the prehabilitation because they were no longer candidates for surgery due to disease progression. After surgery, two died and three withdrew at 12 weeks follow-up. Finally, 15 patients completed the study.

Of the 237 scheduled aerobic and resistance training sessions, 182 were completed, resulting in an overall attendance of 77%, while attendance to IMT was 68%, with 257 sessions completed of the 378 prescribed. No exercise-related adverse events were reported during or after exercise sessions. Twenty subjects completed the satisfaction questionnaire. The median satisfaction score was 9.0 (8.0 to 9.9) on a 10-point scale (Table 1). For all items, the majority of the participants were “satisfied” to “very satisfied”. Responses to the clarity of the internet-based program included “unsatisfied” or “not at all satisfied” for three participants (15%).

### 3.2. Participant Characteristics

Participant characteristics of the enrolled patients are summarized in Table 2. Participants had a mean age of 61.7 ± 10.6 years and included 16 men (70%). Sixteen subjects (70%) received neoadjuvant treatment and started the prehabilitation after its completion, while the remaining seven (30%) were treated in primary surgery and started the exercise program at diagnosis. The mean length of the preoperative periods was 23.7 ± 6.5 days.

### 3.3. Evolution after Intervention

**Figure 2:**
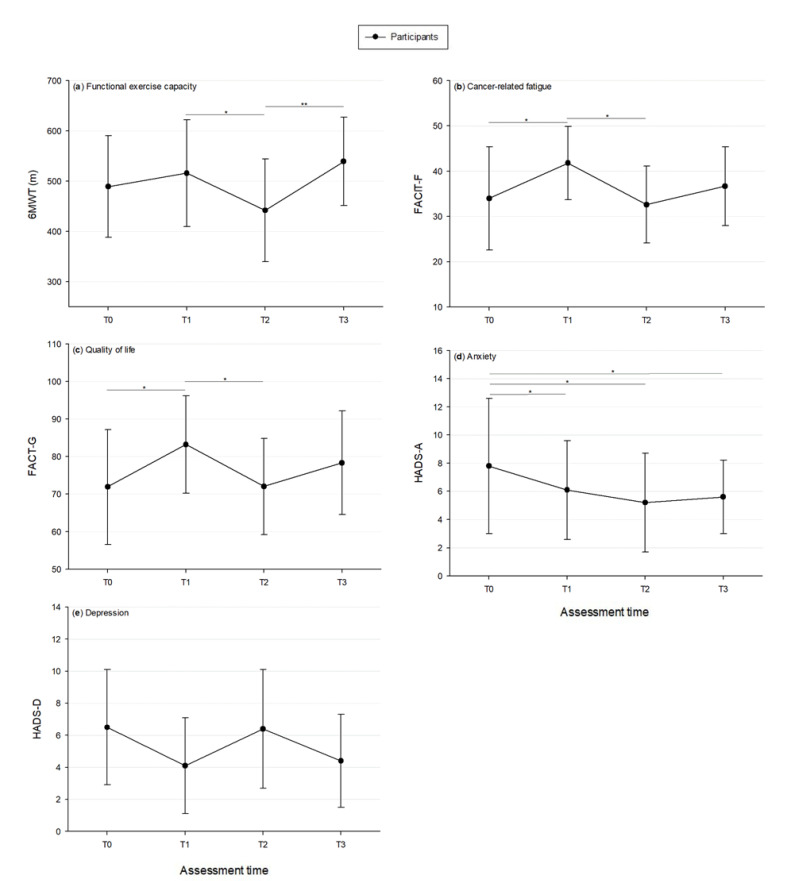
Evolution of (**a**) The 6 min walk test (6MWT); (**b**) The Functional Assessment of Chronic Illness Therapy-Fatigue Scale (FACIT-F); (**c**) The Functional Assessment of Cancer Therapy-General (FACT-G); (**d**) The Hospital Anxiety and Depression Scale-anxiety (HADS-A), and (**e**) The Hospital Anxiety and Depression Scale-depression (HADS-D) throughout the study. T0—at the beginning of the prehabilitation program; T1—at the end of prehabilitation; T2—4 weeks postsurgery; T3—12 weeks postsurgery. Data are mean ± SD. * *p* < 0.05; ** *p* < 0.001

Results of evolution after tele-prehabilitation are provided in Figure 2 and Table 3. Compared to the baseline, significant improvements were observed for the FACIT-F (+7.8 points, 95% CI −0.7 to 16.4, *p* = 0.039), FACT-G (+11.3 points, 95% CI 2.3 to 20.2, *p* = 0.009), PWB (+4.8 points, 95% CI −0.1 to 9.7, *p* = 0.034), EWB (+2.1 points, 95% CI 0.0 to 4.3, *p* = 0.005) and HADS-A (−1.8 points, 95% CI −4.0 to 0.5, *p* = 0.044) after the tele-prehabilitation program. The improvement in the 6MWD between baseline and post-prehabilitation was not statistically significant (+ 26.8 m, 95% CI −0.5 to 54.1, *p* > 0.05) [29]. A significant decline in 6MWD (−74.2 m, 95% CI −143.3 to −5.1, *p* = 0.003), FACIT-F (−9.3 points, 95% CI −18.2 to −0.4, *p* = 0.011), FACT-G (−11.2 points, 95% CI −22.0 to −0.4, *p* = 0.008), PWB (−5.0 points, 95% CI −9.5 to −0.4, *p* = 0.032) and FWB (−4.9 points, 95% CI −9.0 to −0.8, *p* = 0.004) was observed from presurgery to 4 weeks postoperative. A significant improvement in 6MWD (+97.4 m, 95% CI 30.9 to 163.8, *p* < 0.001) and FWB (+3.7, 95% CI 0.3 to 7.1, *p* = 0.042) was shown from 4 to 12 weeks postoperatively. Compared to the baseline, a significant increase in EWB (+2.5 points, 95% CI 0.7 to 4.4, *p* < 0.001) and a significant reduction in HADS-A (−2.6, 95% CI −4.6 to −0.5, *p* = 0.002) were observed 4 weeks after surgery. Twelve weeks after surgery, no differences were observed for the 6MWT, FACIT-F, FACT-G, PWB, SWB, FWB and HADS-D compared to the baseline, while the EWB (+2.3 points, 95% CI 0.3 to 4.3, *p* = 0.003) and HADS-A (−2.2, 95% CI −4.8 to −0.4, *p* = 0.008) scores were significantly improved. There was no significant change in SWB and HADS-D at any time point.

## 4. Discussion

This study investigated the feasibility of a tele-prehabilitation program in esophagogastric cancer patients requiring surgery and the preliminary effects of this program on functional exercise capacity, CRF, QoL, anxiety and depression before surgery and 4 and 12 weeks after surgery.

The findings showed that tele-prehabilitation is feasible, safe, well-accepted and appreciated by esophagogastric cancer patients scheduled for tumor resection. The recruitment rate of eligible patients (96%) was comparable or even better than other studies investigating the effects of prehabilitation in esophagogastric cancer patients [17,18,20].

Ninety-six percent of the enrolled patients completed the prehabilitation program, which is similar to the study of Minnella et al., who also performed a prehabilitation in esophagogastric cancer patients [17]. Several possible reasons can explain the high retention rate of the exercise program. Firstly, the exercise program was home-based with telecommunication support, which enhanced access to the preoperative exercise program by reducing transportation requirements. Of course, patients with cancer often have to go to hospital for further consultation or examination, but adding visits to healthcare providers and facilities can become a burden for the patients who are often elderly and in poor physical condition. Problems related to transportation, including finding parking, paying for parking or arranging transportation, have been reported as the main barrier to participation in prehabilitation by patients [21]. Secondly, exercise programs were tailored based on the patients’ physical fitness and a weekly phone call was made to answer questions the participants had, to motivate them to complete the exercises, and to adapt their program if necessary. Furthermore, the majority of patients had family support to help them use the website if necessary. Advice and support about the importance of preoperative exercise from the surgeon was also very helpful.

The attendance rate was 77% for the aerobic and resistance training, which is higher than previous studies [17,20], while the attendance rate in the IMT sessions was 68%, which is lower than another study [35]. The most frequently reported reasons for missing exercise sessions were pain, lack of motivation and energy, bad weather, and fatigue. Neoadjuvant treatment, received by 70% of patients, is related to short- and long-term side effects, including increased fatigue and impaired physical function [36,37]; therefore, it probably played a role in decreasing attendance to prehabilitation sessions. Consequently, to prevent this decline in patients undergoing neoadjuvant treatment, it might be wiser to implement the exercise program at the time of diagnosis until surgery, rather than during the waiting surgery period at the end of the neoadjuvant treatment. Indeed, the effectiveness of exercise and nutritional interventions to preserve physical fitness and body composition throughout neoadjuvant chemoradiotherapy has been previously reported for esophageal cancer patients [37].

Participant satisfaction in the program was high compared to previous tele-rehabilitation studies [38,39]. Although the majority of patients reported to be very satisfied with all items, the clarity of the internet-based program was the only item for which three patients were unsatisfied. Some technical problems with the videos were reported and the interface of the website does not seem to be the most suitable for technology novices. Similar to this finding, Doiron-Cadrin reported excellent satisfaction toward tele-prehabilitation in patients awaiting arthroplasty but only 36% of the patients found the use of the application easy [38].

This study showed preliminary results regarding the possible effectiveness of a tele-prehabilitation in esophagogastric cancer patients scheduled for tumor resection. Physical prehabilitation aims to enhance preoperative functional capacity to prepare patients for surgery and have a positive impact on postoperative outcomes [40]. The 6MWT has been shown to be useful to evaluate preoperative physical status before esophageal resection [41]. A 6MWD ≤ 454 m has been identified as a threshold for predicting postoperative Clavien–Dindo grade ≥ II complications after esophagectomy [41]. In this trial, the mean 6MWD at baseline was 489 ± 101 m (90% of the predicted 6MWD), with nine patients (39%) scoring less than 454 m. After prehabilitation, the 6MWD was increased by 27 m. This enhancement was not significant but was greater than what is perceived by patients undergoing abdominal surgery as clinically relevant and may pave the way for designing future trials [29]. Therefore, although not significant, this result made a positive and noticeable improvement to patients. The number of patients with a 6MWD below the cut-off of 454 m was six after prehabilitation. The mean 6MWD followed a clinical and significant decrease from presurgery to 4 weeks postsurgery, followed by a clinical and significant increase from 4 to 12 weeks postsurgery. The mean distance walked was not different 12 weeks after surgery compared to the baseline. These findings are similar to Guinan et al., who reported the same progression of the 6MWD one and six months after esophagectomy but without prehabilitation [42]. In contrast to our study, Taguchi et al. aimed to observe the evolution of exercise capacity without a preoperative exercise program following open surgery, and showed that the maximum oxygen uptake was significantly decreased three months after surgery compared to presurgery [43].

CRF is a prevalent and distressing symptom experienced by a high proportion of the cancer population [44]. Exercise programs have been reported to be effective for the management of CRF [45]. We found that the tele-prehabilitation program followed a CRF improvement before surgery, with an increase of 7.8 points, which is perceived as clinically relevant [31]. Few studies have investigated the effect of prehabilitation on CRF. Santa Mina et al. reported no difference in CRF before surgery between prehabilitation and control groups in men undergoing radical prostatectomy [46]. This difference in results between the two studies may be explained because the majority of the patients in our study received neoadjuvant treatment before starting prehabilitation, which is proven to induce a decline in CRF. Indeed, the CRF score at baseline was already deteriorated, with a mean score of 34 points out of 52. Patients with higher baseline fatigue may be more likely to improve after an exercise program, as reported with functional capacity before and after colorectal cancer surgery [47].

It has been shown that patients experience a deterioration in QoL as a consequence of the short- and long-term side effects of esophagogastric surgery [7]. In the present study, the mean FACT-G at baseline was 72 points out of 102 and comparable to other cancer studies [48,49]. The QoL total score increased significantly by 11.3 points after the tele-prehabilitation program and reached the MCID of 4.0 previously reported as clinically relevant [50]. This is similar to a previous study, in which the prehabilitation group’s QoL total score improved after four weeks of prehabilitation before liver resection [51]. This positive effect on QoL may be explained in part by the improvement of CRF after prehabilitation. Furthermore, by a proactive approach, the participation in the exercise program may play a role in improving the QoL by making patients active in their care. It is interesting to note that emotional well-being followed an enhancement from baseline to presurgery and this improvement was maintained 4 and 12 weeks postsurgery.

Prevalence of psychological distress, including anxiety and depression, is very high in cancer patients [52]. In the context of surgery, it is of clinical interest to reduce or prevent this psychological distress before major surgery because it is associated with longer hospital stays, higher morbidity and mortality rates [53,54], slowing down of the healing process, and higher levels of pain and non-compliance with medical treatment [55,56]. In the present study, depression did not significantly change at any time point. Regarding anxiety, we observed a significant reduction in anxiety from baseline to post-prehabilitation, whereas in general, in the absence of preoperative intervention, most patients awaiting surgery experience anxiety because they perceive surgery as a threat to their lives [57]. Furthermore, anxiety scores were also significantly reduced 4 and 12 weeks postoperatively compared to baseline. In contrast, a study exploring the natural evolution of anxiety before and after surgery in esophageal cancer patients showed that anxiety symptoms remained stable over time [58].

As the length of the tele-prehabilitation program was time-limited to two weeks for patients treated in primary surgery in our hospital, the length of the program was set at four weeks for patient receiving neoadjuvant treatment to reduce heterogeneity among participants. However, this two week duration may be insufficient to be effective before esophagogastric surgery, and in the absence of evidence of its effectiveness, a longer duration, in accordance with national cancer treatment targets, could be considered without compromising disease-free survival and overall survival. In addition, a high intensity during exercise training may be more effective to observe the benefits of tele-prehabilitation than moderate-intensity training. In a recent study, eight sessions of high-intensity interval training over three weeks increased aerobic and exercise capacities in lung cancer patients awaiting surgery [59]. Furthermore, the comparison of our findings to a control group could have highlighted an effect of tele-prehabilitation before surgery on the different parameters measured.

Limitations of our study include the heterogeneity among participants in terms of types of cancer, neoadjuvant treatment, type of surgery and duration of prehabilitation. In addition, the small sample size, single-center design and the lack of a control group limit the interpretation of our findings and may explain some of the conflicting data. However, the heterogeneity and the single-group design were considered appropriate, since the primary objective was to investigate the feasibility of a tele-prehabilitation program. Our study paves the way for randomized controlled trials aiming to assess the effectiveness of a tele-prehabilitation in esophagogastric cancer patients. Furthermore, a comparison between a two-week and four-week program should be investigated in a larger study to determine the optimum duration of prehabilitation in esophagogastric cancer patients. Finally, the prehabilitation program was only composed of a physical component. It could be interesting to combine exercise, nutritional and psychological interventions to determine the benefits of a multimodal tele-prehabilitation in esophagogastric cancer patients undergoing surgery.

## 5. Conclusions

This study demonstrated the feasibility of a tele-prehabilitation among esophagogastric cancer patients undergoing surgery, with a high recruitment rate, retention rate and satisfaction, a good attendance to exercise sessions, and no exercise-related adverse events.

## Figures and Tables

**Table 1:** Results of the satisfaction questionnaire (*n* = 20).

How Satisfied Are You With	Very Satisfied	Satisfied	Somewhat Satisfied	Unsatisfied	Not at All Satisfied
The physiotherapist availability	17 (85.0)	2 (10.0)	1 (5.0)	0 (0.0)	0 (0.0)
The information brochure	11 (55.0)	8 (40.0)	1 (5.0)	0 (0.0)	0 (0.0)
The clarity of the internet-based program	10 (50.0)	6 (30.0)	1 (5.0)	2 (10.0)	1 (5.0)
The program duration	16 (80.0)	3 (15.0)	1 (5.0)	0 (0.0)	0 (0.0)
The program intensity	13 (65.0)	5 (25.0)	2 (10.0)	0 (0.0)	0 (0.0)
Your motivation	9 (45.0)	8 (40.0)	3 (15.0)	0 (0.0)	0 (0.0)
The progress you have made	11 (55.0)	7 (35.0)	2 (10.0)	0 (0.0)	0 (0.0)
Your participation in the program	14 (70.0)	4 (20.0)	2 (10.0)	0 (0.0)	0 (0.0)
How would you rate the overall program on a scale of 0 to 10 (0 = very poor, 10 = excellent)? 9.0 (8.0 to 9.9)					

Data are number (%) and median (interquartile range).

**Table 2:** Participant characteristics.

Variables	Participants
Baseline characteristics (*n* = 23)	
Age (years)	61.7 ± 10.6
Men	16 (69.6)
Weight (kg)	75.0 ± 18.2
Height (cm)	170.6 ± 8.0
BMI (kg/m^2^)	25.6 ± 4.7
ASA classification, II/III	10 (43.5)/13 (56.5)
Smoking status, current/former/never	4 (17.4)/9 (39.1)/10 (43.5)
Drinking status, current/former/occasional/never	7 (30.4)/5 (21.7)/6 (26.1)/5 (21.7)
High blood pressure	15 (65.2)
Cardiac disease	7 (30.4)
Respiratory disease	6 (26.1)
Diabetes mellitus	3 (13.0)
DASI total score	38.9 ± 16.5
6MWT (% pred)	89.5 ± 19.0
FEV1/VC (% pred)	93.4 ± 12.8
Creatinin (mg/dL)	0.8 ± 0.2
Albumin (g/L)	44.3 ± 2.3
Hemoglobin (g/dL)	12.5 ± 1.3
C-reactive protein (mg/L)	3.3 ± 2.3
Tumor location, oesophagus/GE junction/gastric	16 (69.5)/3 (13.0)/4 (17.4)
Adenocarcinoma/squamous carcinoma	15 (71.4)/6 (28.6)
Neoadjuvant treatment, CT/CRT	3 (13.0)/13 (54.2)
Postoperative outcomes (*n* = 21)	
AJCC pStage, 0/I/II/III	2 (9.5)/9 (42.9)/4 (19.0)/6 (28.6)
Surgical procedure, three-field esophagectomy/two-field esophagectomy/gastrectomy	5 (23.8)/12 (57.1)/4 (19.0)
Length of hospital stay (days)	14.7 ± 9.5
Clavien-Dindo complications, 1/2/3/4/5	2 (9.5)/8 (38.1)/2 (9.5)/0 (0.0)/2 (9.5)
Readmissions, 30 days/90 days	1 (4.8)/1 (4.8)
In-hospital mortality	2 (9.5)

Data are mean ± SD and numbers (percentage) as appropriate. % pred—percentage of predicted value; 6MWT—6 min walk test; AJCC—American Joint Committee on Cancer; ASA—American Society of Anesthesiologists; BMI—body mass index; CRT—chemoradiotherapy; CT—chemotherapy; DASI—Duke Activity Status Index; FEV1—forced expiratory volume in one second; GE—gastroesophageal; VC—vital capacity.

**Table 3:** Evolution after tele-prehabilitation on functional exercise capacity, cancer-related fatigue, quality of life, anxiety and depression in esophagogastric cancer patients.

Variables	T0 (*n* = 23)	T1 (*n* = 20)	T2 (*n* = 18)	T3 (*n* = 15)	*p*-Value ^¥^
6MWT (m)	489.1 ± 101.1	515.9 ± 106.4 ^(T2)^	441.7 ± 102.2 ^(T1, T3)^	539.1 ± 87.8 ^(T2)^	<0.001
FACIT-F	34.0 ± 11.4 ^(T1)^	41.8 ± 8.1 ^(T0, T2)^	32.6 ± 8.5 ^(T1)^	36.7 ± 8.7	0.010
FACT-G	71.9 ± 15.3 ^(T1)^	83.2 ± 13.0 ^(T0,T2)^	72.0 ± 12.8 ^(T1)^	78.3 ± 13.8	0.003
PWB	19.5 ± 7.0 ^(T1)^	24.4 ± 3.0 ^(T0, T2)^	19.4 ± 5.5 ^(T1)^	21.3 ± 5.3	0.019
SWB	20.4 ± 4.9	22.4 ± 4.6	20.7 ± 4.7	21.6 ± 3.6	0.061
EWB	16.4 ± 4.1 ^(T1, T2, T3)^	18.6 ± 3.4 ^(T0)^	19.0 ± 3.6 ^(T0)^	18.7 ± 2.9 ^(T0)^	<0.001
FWB	15.6 ± 6.4	17.9 ± 6.3 ^(T2)^	13.0 ± 4.2 ^(T1, T3)^	16.7 ± 4.6 ^(T2)^	0.005
HADS-A	7.8 ± 4.8 ^(T1,T2,T3)^	6.1 ± 3.5 ^(T0)^	5.2 ± 3.5 ^(T0)^	5.6 ± 2.6 ^(T0)^	0.001
HADS-D	6.5 ± 3.6	4.1 ± 3.0	6.4 ± 3.7	4.4 ± 2.9	0.010 ^#^

Data are mean ± SD. 6MWT—6-min walk test; EWB—emotional well-being; FACIT-F—functional assessment of chronic illness therapy-fatigue; FACT-G—functional assessment of cancer therapy-general; FWB—functional well-being; HADS-A—hospital anxiety and depression scale-anxiety; HADS-D—hospital anxiety and depression scale-depression; PWB—physical well-being; SWB—social well-being; T0—at the beginning of the prehabilitation program; T1—at the end of prehabilitation; T2—4 weeks postsurgery; T3—12 weeks postsurgery. ¥—Repeated-measures analysis of variance. ^#^ The repeated-measures analysis of variance showed a significant difference in HADS-D changes over the four time points but no significant differences were observed after Holm–Sidak post hoc corrections. (T0), (T1), (T2), (T3): Statistically significant differences (*p* < 0.05) between time points from Holm–Sidak post hoc corrections are indicated by the respective abbreviations of the different time point in individual columns.
